# Case report: Synchronous tumors of the female reproductive tract in systemic lupus erythematosus: report of two cases and review of the literature

**DOI:** 10.3389/fonc.2024.1322598

**Published:** 2024-02-21

**Authors:** Ling Wang, Qin Zhang, Nan Shi, Jiaxi Wang, Shuang Song, Huadi Yang, Xingbei Chen

**Affiliations:** ^1^ Department of Gynecology and Obstetrics, The First Affiliated Hospital of Zhejiang Chinese Medical University (Zhejiang Provincial Hospital of Chinese Medicine), Hangzhou, China; ^2^ Department of TCM Gynecology, Hangzhou TCM Hospital Affiliated to Zhejiang Chinese Medical University, Hangzhou, China; ^3^ Department of Pathology, The First Affiliated Hospital of Zhejiang Chinese Medical University (Zhejiang Provincial Hospital of Chinese Medicine), Hangzhou, China

**Keywords:** synchronous tumors, female reproductive tract tumors, systemic lupus erythematosus, vulvar cancer, vaginal cancer, cervical cancer, case report

## Abstract

**Background:**

Systemic lupus erythematosus (SLE) is a chronic autoimmune disease that affects multiple systems. Patients with SLE are prone to a variety of malignancies, especially neoplasms of the female reproductive tract. Synchronous tumors, considered to involve multiple sites, are rare in the female reproductive tract. There are hardly any reports of SLE with synchronous reproductive tract tumors.

**Case presentation:**

We report the occurrence of two to three reproductive tract tumors in two women with SLE. A 52-year-old woman was diagnosed with vulvar cancer and cervical cancer. Another woman, aged 67, was diagnosed with concurrent vulvar cancer, vaginal cancer, and cervical cancer and also presented with a suspected lung cancer.

**Conclusion:**

The presence of synchronous tumors of the reproductive tract in patients with SLE is uncommon and can be easily disregarded. It is crucial to highlight that SLE patients with multiple primary malignancies exhibit notable late-stage presentation at the time of diagnosis, inadequate disease-free survival, poor overall survival, rapid progression rates, and mortality. Consequently, greater awareness must be raised regarding synchronous reproductive tract tumors in patients with SLE. Regular comprehensive cancer screening and management should be implemented for individuals diagnosed with SLE.

## 1 Background

Systemic lupus erythematosus (SLE) is a prevalent chronic multisystem autoimmune disorder that impacts 3.41 million individuals globally, with a significantly higher prevalence among women (10:1 female-to-male ratio) (1). The standard treatment for SLE includes glucocorticoids and immunosuppressants. Studies have confirmed an association between SLE and various malignancies (2), such as breast cancer, lung cancer, and Hodgkin’s lymphoma (3–5). In addition, SLE is a risk factor for neoplasms of the female reproductive tract. It has been reported that cervical and vulvar cancers are more common in patients with SLE than in the general population (6, 7). Synchronous tumors, which encompass multiple locations, are infrequent in the female reproductive tract, only comprising a mere 0.6%–5.4% of all tumors of the female genital system (8). Vulvar and cervical cancers occur infrequently concurrently, whereas endometrial and ovarian cancers are the most prevalent synchronous tumors (8). There are almost no reports of SLE in conjunction with synchronous tumors of the reproductive tract, despite the fact that the association between SLE and malignancies is becoming increasingly acknowledged.

In this study, we report the concurrent development of malignancies in the reproductive tract of two patients with SLE. Since being diagnosed with SLE more than two decades ago, both individuals have been taking prednisone. Vulvar carcinoma and cervical carcinoma were identified in the first patient, a 52-year-old woman. The second patient, a 67-year-old woman, was diagnosed with vulvar, vaginal, and cervical cancers, along with a suspected case of lung cancer. This study aimed to demonstrate that women with SLE may have a higher risk of reproductive tract tumors, even synchronous ones. Therefore, appropriate screening and attention should be given to these patients.

## 2 Case presentation

### 2.1 Case no. 1

A 52-year-old postmenopausal married woman presented to our outpatient department in June 2022 with a “vulvar mass found for over 7 years, with pain for 3 years.” She had a history of SLE and was first diagnosed in 1989. In the first year, she took oral prednisone acetate (Prednisone) tablets (40 mg, qd), but later decreased (7.5 mg, qd), and continued to take them. From 2021, dispersible mycophenolate mofetil (CellCept®) tablets (0.5 g, qd) and hydroxychloroquine sulfate (Plaquenil®) tablets (0.2 g, bid) were added. She also had hypertension and was on 1# of irbesartan once a day.

**Figure 1:**
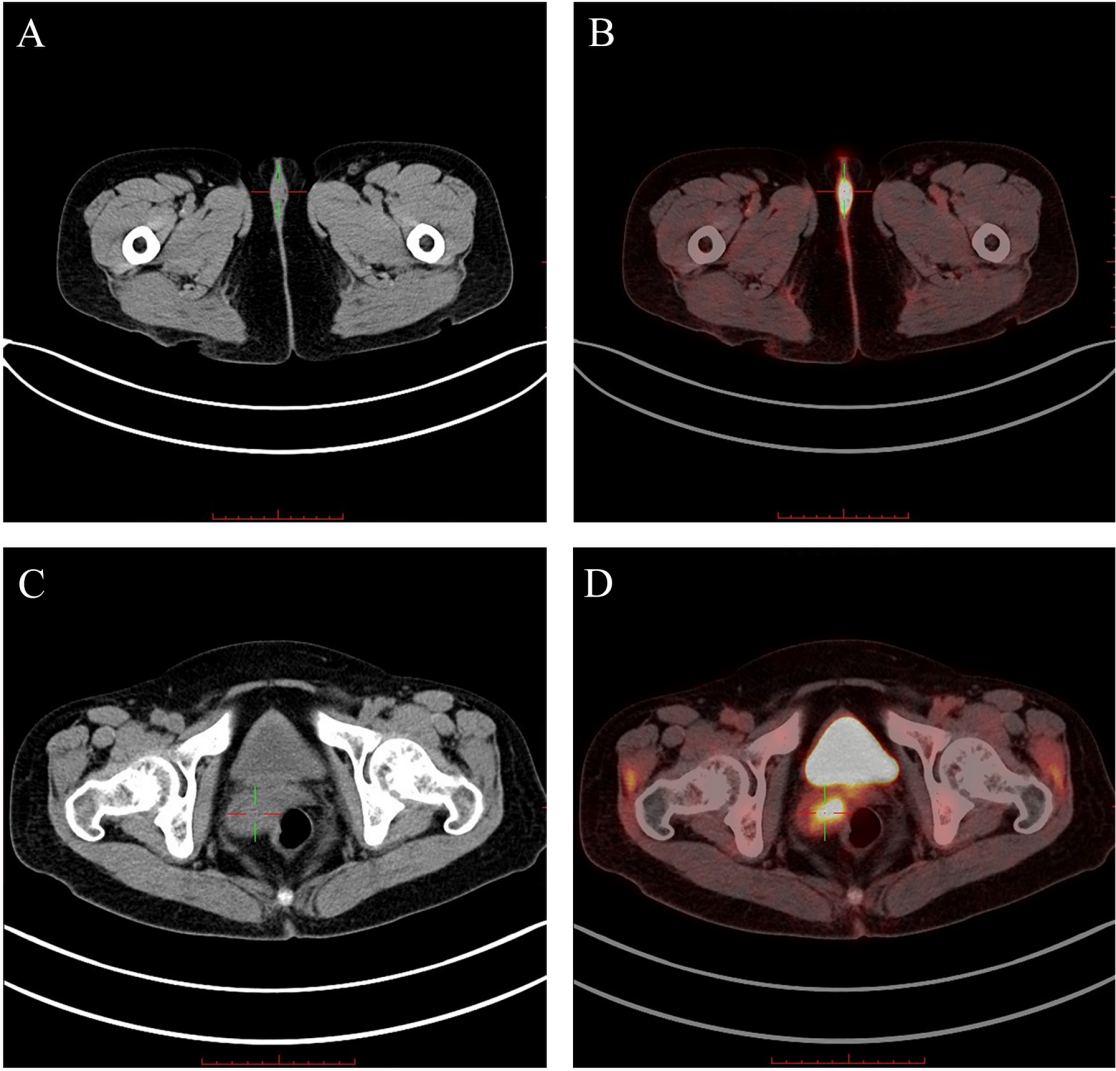
The PET-CT scans obtained from case 1 ("+" presents the vulvar/cervical lesion): **(A)** The CT showed a low-density nodular shadow in the vulva. **(B)** The PET-CT showed significantly increased 18F-FDG metabolism in the vulvar lesion. **(C)** The CT showed an uneven density mass shadow in the cervix. **(D)** The PET-CT showed significantly increased 18F-FDG metabolism in the cervical lesion.18F-FDG, 18F-fluorodeoxyglucose.

Specialist examination revealed adhesions of the left labia majora, with a 3.0 cm × 3.0 cm × 1.5 cm mass. The skin over the mass had hypopigmentation and localized ulceration, with localized white lesions on both labia minora. The vagina was patent, with a 2.5 cm × 2.0 cm × 1.0 cm gray-white mass at the cervical labium posterius and blood was present on touch by medical cotton swab. The uterus was in retroversion, of normal size, with no abnormalities in the adnexa bilaterally. Laboratory tumor markers showed squamous cell carcinoma antigen (SCCA) at 2.6 ng/ml. Positron emission tomography-computed tomography indicated a cervical mass (1.8x2.8cm) and a vulvar mass (2.4x1.0cm) with significantly increased metabolism of 18F-fluorodeoxyglucose, suggestive of cervical cancer (**Figures 1 A-D**). Human papillomavirus (HPV)16 was positive, and the thinprep cytologic test (TCT) was negative for intraepithelial lesions or malignancy. Cytology DNA presented one or two aneuploid abnormal cells. Biopsy pathology of the cervical and vulvar masses indicated vulvar *in situ* squamous carcinoma and squamous cell carcinoma (SCC) of the cervix.

**Figure 2:**
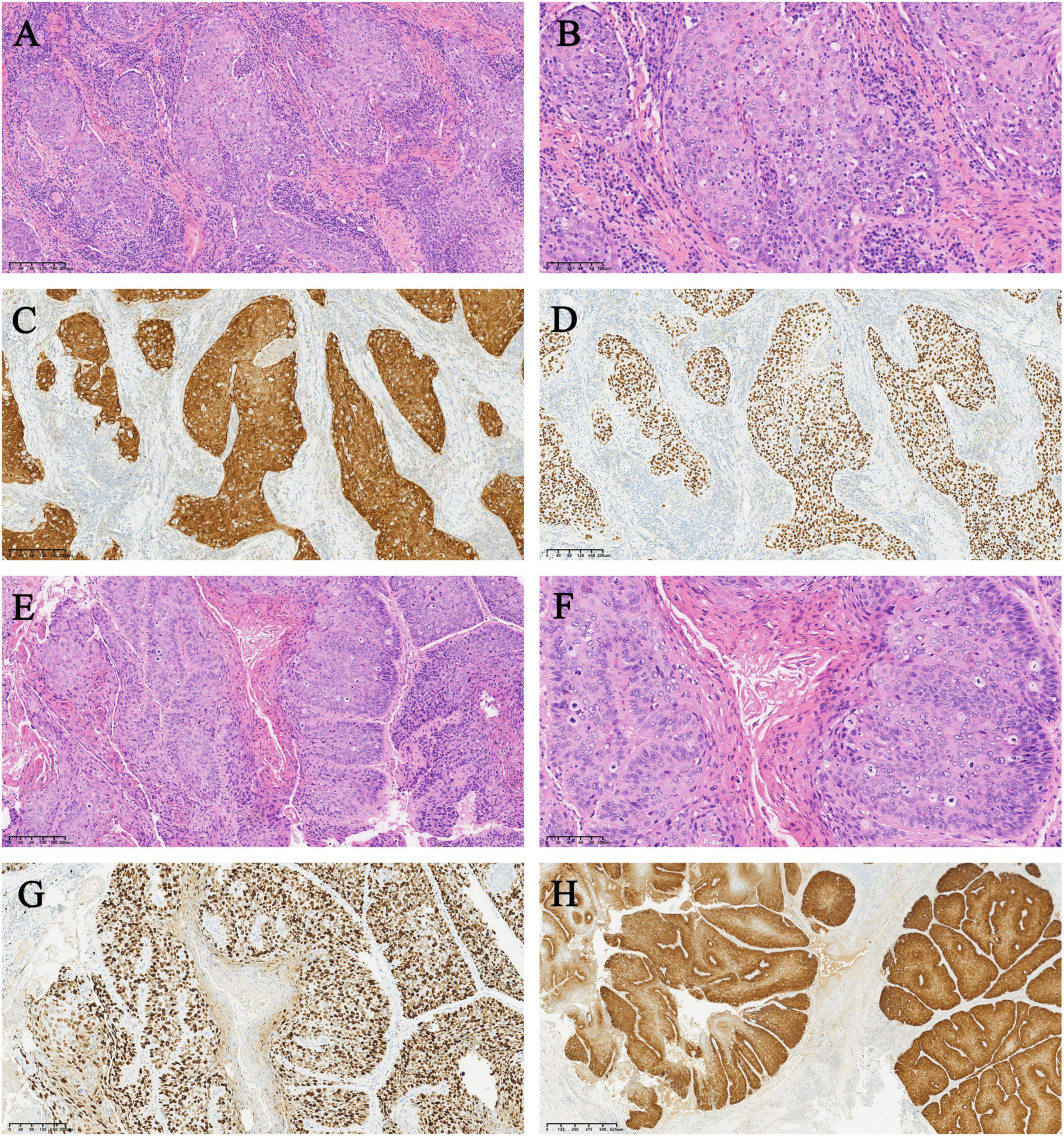
Characteristics of the cervical squamous cell carcinoma and vulvar squamous cell carcinoma for case 1. **(A)** The cervical tumor cells were arranged in a nest-like pattern, with obvious cell abnormalities. **(B)** The cervical tumor cells were polygonal and the nuclei were round or oval. The mitotic appearance was easy to see, and no keratinized beads were observed. **(C)** P16 was diffuse positive in the cervical tumor cells. **(D)** P40 was diffuse positive in cervical tumor cells. **(E)** The vulvar tumor cells were arranged in a nested pattern, invading the stroma. **(F)** Keratinocytes were visible in vulvar tumor cells. **(G)** Ki-67 was approximately 90% positive in vulvar tumor cells. **(H)** P16 was diffusely positive in vulvar tumor cells.

Ultimately, the patient underwent transabdominal radical hysterectomy, pelvic lymphadenectomy, pelvic adhesion release, para-abdominal aortic lymphadenectomy, bilateral salpingo-oophorectomy, local radical vulvectomy and bilateral inguinal lymph node dissection. Histologically, the cervix showed moderately differentiated SCC with nest-like arrangements of cells (**Figures 2A, B**), significant cellular atypia, numerous mitotic figures, and lymph-vascular space invasion. Cancer tissue invaded approximately half of the cervical wall but did not reach the internal os, with high-grade squamous epithelial lesions visible in the vaginal wall, negative surgical margins on the anterior and posterior vaginal walls, negative parametria bilaterally, and negative blood vessels in both ovaries. Vulvar mass showed high- to moderately differentiated SCC (**Figures 2E, F**): tumor with nest-like arrangements, marked cellular atypia, numerous mitotic figures, keratin pearl formation, invasive growth of the tumor, high-grade squamous intraepithelial lesion (HSIL) in the surrounding rough area, and negative surgical margins around the skin and at the base. Immunohistochemistry indicated that the tumor cells were positive for epithelial markers CK5/6 and P40 (**Figure 2D**). The ki-67 index of cervical tumors was 70%, and that of vulvar tumors was 90% (**Figure 2G**). The expression of P53 was less than 5%, indicating that the tumor was wild-type. The strong positive expression of P16 proved that the tumor was HPV-related (**Figures 2C, H**). The blood vessels and lymphatic vessels were labeled with CD34 and D2-40, which showed vascular invasion of the tumor.

### 2.2 Case no. 2

A 67-year-old postmenopausal woman with a history of vulvar ulcers and pain for 3 years presented for consultation in July 2022. She was diagnosed with SLE in 1997. At the beginning of 3 months, she took prednisone (10 mg, bid), hydroxychloroquine (0.2 g, po bid), and cyclophosphamide injection (the dose and period were not known). Afterward, she felt that the medical treatment was troublesome; therefore, she continued the use of prednisone but stopped taking the other medications. Prednisone gradually decreased in dosage to 7.5 mg (qd) for the long term. Due to leukopenia, she had been prescribed leucogen tablets (40 mg, qd). In addition, she suffered from hypertension and took an antihypertensive medication intermittently.

The gynecological examination revealed a mass at the posterior commissure of the labia, with a keratinized and ulcerated surface showing hypopigmentation, measuring approximately 3.0 cm × 2.0 cm on the left and 1.5 cm ×2.0 cm on the right, adjacent to the vaginal orifice. With the aid of a speculum placed vaginally, the cervix was exposed, showing cervical atrophy and ulcerative changes at the 10 o’clock position, with punctate congestion at the 2–4 o’clock positions on the vaginal wall, but without obvious tumorous protrusions. The uterus was atrophic, with no abnormalities in the adnexa bilaterally. HPV testing was positive for HPV33/53/82, while cervical cytology DNA showed more than three aneuploid abnormal cells. TCT was ASC-H. Further biopsies revealed HSIL/VIN III in the vaginal mucosa. The cervical mucosa showed HSIL/CIN III, suspicious for microinvasion. The right labia minora mucosa showed HSIL/VIN III with microinvasion. The results of her CT target reconstruction evaluation indicated a solid ground-glass nodule in the upper lobe of the right lung. Compared to the results from January 18, 2020, it can be seen that the ground-glass portion was slightly larger and the solid portion was similar. A specialized consultation in the pulmonary nodule outpatient department was needed. Specialist physicians thought that the possibility of a malignancy could not be ruled out, and surgical resection was recommended.

**Figure 3:**
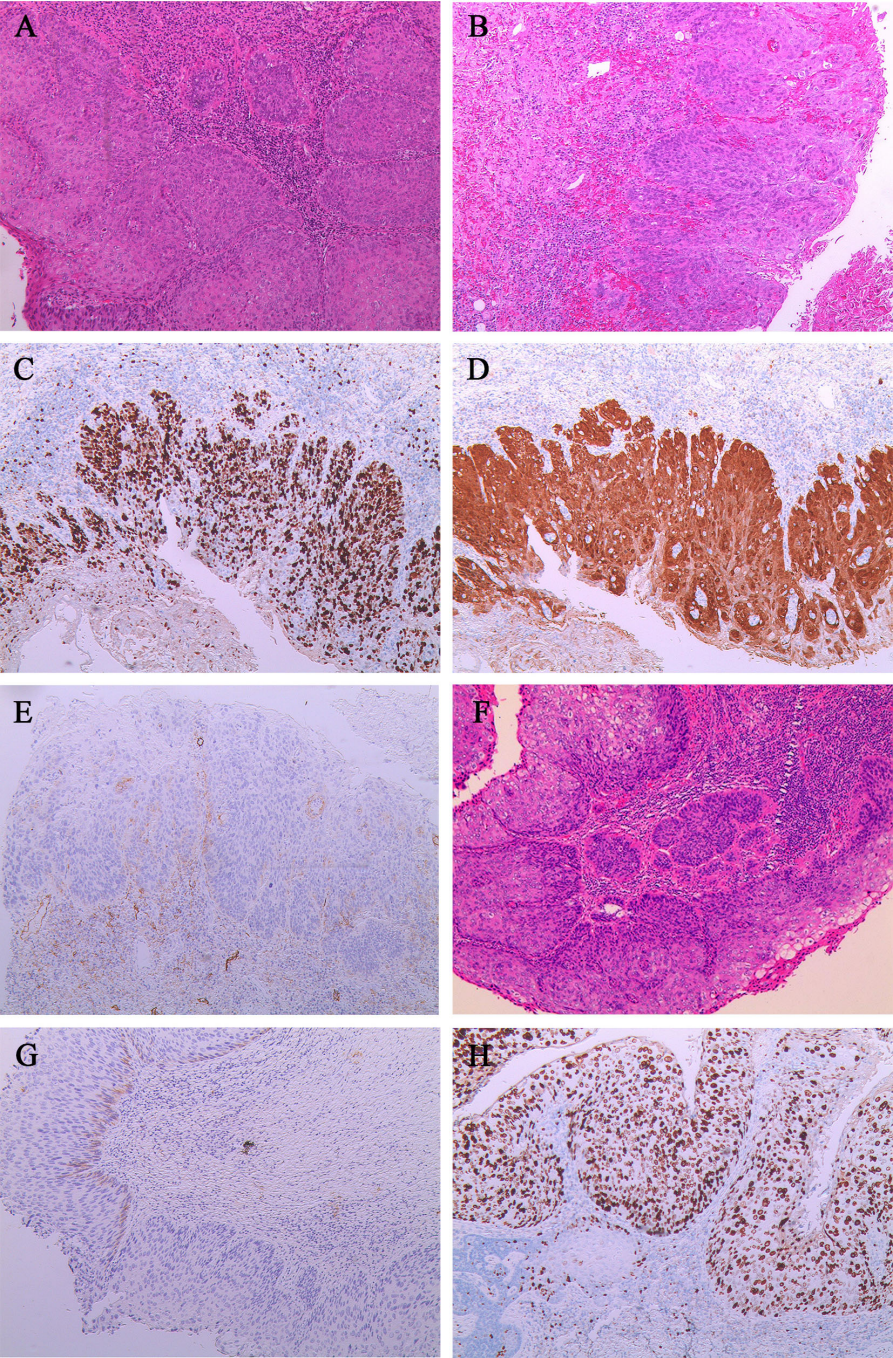
Characteristics of the cervical squamous cell carcinoma, vulvar squamous cell carcinoma, and vaginal squamous cell carcinoma for case 2. **(A)** The cervix was HSIL/CIN III with microinvasion (stroma invasion depth, <0.3cm).**(B)** HSIL in the vulva with focal invasion (stroma invasion depth, <0.1cm). **(C)** The Ki-67 was about 90% positive in vulvar tumor cells.**(D)** The P16 was diffusely positive in vulvar tumor cells. **(E)** The D2-40 is negative in the partial basal layer of the vulvar squamous epithelium at the microinvasion.**(F)** HSIL in the vaginal wall with focal invasion (stroma invasion depth, <0.1cm).**(G)** The D2-40 was negative in the partial basal layer of the vaginal squamous epithelium at the microinvasion. **(H)** The Ki-67 epithelial was 90% positive in vaginal tumor cells.

Under laparoscopy, inguinal lymph node dissection, pelvic lymphadenectomy, pelvic adhesion lysis, bilateral salpingo-oophorectomy, transvaginal modified radical hysterectomy, vaginectomy, and local radical vulvectomy were performed. Histology suggested the cervix was HSIL/CIN III with microinvasion, invading the stroma. (**Figure 3A**). Atypical cell polarity was disordered, reaching the upper one-third of the squamous epithelial layer. HSIL with focal invasion was noted in the vulva and the vagina (**Figures 3B, F**), with no lymph node metastasis detected. The immunohistochemistry results showed tumor cells positive for P16, Ki-67, EGFR, MLH1, PMS2, MSH2, and MSH6. P16 showed diffuse strong positivity (**Figure 3D**), and the Ki-67 positive rate was 90% (**Figures 3C, H**). However, negative staining for D2-40 was observed in the partial area of the basal lamina (**Figures 3E, G**).

### 2.3 Outcome and follow-up

Based on the clinical and pathological results, both patients were considered to have synchronous tumors of the reproductive tract. The first patient was diagnosed with the following: 1) vulvar malignancy (stage IB); 2) cervical malignancy (stage IB2); and 3) high-grade squamous lesion of the vaginal wall. The second was diagnosed with 1) vulvar malignancy (stage IB); 2) vaginal malignancy (stage I); and 3) cervical malignancy (stage IA1). The patient in case 1 received intensity-modulated radiation therapy using 6-MV X-rays, with a planned dose of 45 Gy delivered in 25 fractions. However, due to acute appendicitis, only one fraction was administered. Case 2 did not undergo postoperative radiotherapy and chemotherapy. Moreover, she was diagnosed with bipolar disorder caused by SLE, with intermittent use of Depakin and psychotherapy. Therefore, she and her family did not seek surgical treatment for pulmonary nodules. At the end of a 1-year follow-up period, neither of the two patients showed any signs of tumor recurrence or metastasis.

## 3 Literature review

Only one English-language case of synchronous lesions in the reproductive tract among women with SLE was found through a systematic PubMed search. A summary of the key characteristics of this report, in addition to our two cases, is provided in **Table 1**.

**Table 1:** Features of synchronous reproductive tract tumors in female systemic lupus erythematosus (SLE) patients.

Author and citation	Age (years)	Basic disease	Details of SLE				Details of tumors								Follow-up
			Age of onset	Clinical symptoms	Laboratory examination	Treatment	Age of onset (years)	Sites involved	Clinical symptoms	Laboratory examination	Gross figure	Histopathological examination	Surgical treatment	Other treatment	
Stepanić et al. (9)	27	NA	13	Non-erosive arthritis and glomerulonephritis	Thrombocytopenic, ANA (+), anti-dsDNA (+)	From 13 to 18 years old: glucocorticoids, cyclophosphamide From 18 to 27 years old: methylprednisolone, 8–16 mg (po qd); hydroxychloroquine, 150 mg (po qd)	25	Cervix; vulva	Vulva pruritus	Blood test: no aberrant, HPV: (−), TCT: LSIL	Vulva leukoplakia	Cervical LSIL; vulvar grade I intraepithelial neoplasiaaccompanied by leukocytosis	NA	5-Fluorouracil ×1 month (chemotherapy); imiquimod, (biw) ×2 months; flumethasone-neomycin (qd) ×1 month	No recurrence
This study, case 1	52	Hypertension	19	Oliguria	NA	From 1989 to 1990: prednisone, 40 mg (po qd) From 1991 to 2020: prednisone, 7.5 mg (po qd) From 2021 to 2023: prednisone, 7.5 mg (po qd); dispersible mycophenolate mofetil, 0.5 g (po qd); hydroxychloroquine, 0.2 g (po bid)	52	Cervix; vulva	Vulvar mass and pain	SCCA: 2.6 ng/ml HPV: 16 (+) TCT: NILM	1) Cervical gray-white mass (2.5 cm × 2.0 cm × 1.0 cm) 2) Vulvar gray-white mass (3.0 cm × 3.0 cm × 1.5 cm)	Cervical moderately differentiated squamous cell carcinoma; vulvar highly to moderately differentiated squamous cell carcinoma	Transabdominal radical hysterectomy, pelvic lymphadenectomy, pelvic adhesion release, para-abdominal aortic lymphadenectomy, bilateral salpingo-oophorectomy, local radical vulvectomy, and bilateral inguinal lymph node dissection	Radiation therapy: IMRT with 6-MV X-ray (a total of 1.8 Gy/1 F)	No recurrence after 1 year
This study, case 2	67	Hypertension	42	Fever, ascites, cheek erythema	NA	First 3 months: prednisone, 10 mg (po bid); hydroxychloroquine, 0.2 g (po bid); cyclophosphamide injection Later: prednisone, 7.5 mg (po qd); leucogen tablets, 40 mg (po qd)	67	Cervix; vulva; vagina	Vulvar ulcer and pain	CA-125: 37.7 U/ml; SF: 244 ng/ml; HPV: 33/53/82 (+); TCT: ASC-H	Vulvar mass (left, 3.0 cm × 2.0 cm; right, 1.5 cm × 2.0 cm)	Cervical HSIL involving glands with focal infiltration Vaginal HSIL with focal infiltration Vulvar HSIL with focal infiltration	Laparoscopic inguinal lymph node dissection, pelvic lymphadenectomy, pelvic adhesion lysis, bilateral salpingo-oophorectomy and transvaginal modified radical hysterectomy, vaginectomy, local radical vulvectomy	No chemotherapy or radiotherapy	No recurrence after 1 year

ANA, anti-nuclear antibody; dsDNA, double-stranded deoxyribonucleic acid; WBC, white blood cell; SCCA, squamous cell carcinoma antigen; CA-125, carbohydrate antigen 125; SF, serum ferritin; HPV, human papillomavirus; TCT, ThinPrep cytology test; ASC-H, atypical squamous cells (cannot exclude high-grade squamous intraepithelial lesion); LSIL, low-grade squamous intraepithelial lesion; HSIL, high-grade squamous intraepithelial lesion; NILM, negative for intraepithelial lesion or malignancy; IMRT, intensity-modulated radiation therapy; NA, not available. (+), positive; (-), negative.

A 13-year-old girl was diagnosed with SLE and had been undergoing prolonged methylprednisolone and hydroxychloroquine treatment. At the age of 27, she sought medical attention for a year-long episode of vulvar irritation. A routine Pap smear for cervical cytology revealed the presence of low-grade squamous intraepithelial lesions. Histopathological analysis of the vulvar biopsy and colposcopy confirmed the presence of grade I intraepithelial neoplasia accompanied by leukocytosis. A month after receiving 5-fluorouracil chemotherapy, the patient discontinued treatment due to an intolerance to the drug.

Therefore, it is indisputable that the co-occurrence of SLE and reproductive tract malignancy is exceedingly uncommon. Patients with SLE may present with malignancies affecting the reproductive tract and other locations. In such circumstances, failure to detect these malignancies early may result in an unfavorable prognosis.

## 4 Discussion

SLE affects multiple systems and is classified as an autoimmune inflammatory connective tissue disease. Although early detection and advanced treatment have greatly improved the survival rate of patients with SLE, malignancy remains a major cause of mortality in these patients, and its incidence is still increasing (10). Many studies have indicated a certain correlation between patients with SLE and reproductive tract tumors (11), which results in a lower survival rate (12). As co-cancer of the reproductive tract is exceedingly uncommon, no reports have indicated an increased risk in patients with SLE. However, a multitude of studies have highlighted the substantial elevation in the incidence and risk of cervical cancer among individuals with SLE compared to the general populace. A retrospective study involving 21,016 patients with SLE showed that the incidence rate of cervical cancer was 0.729, with an odds ratio (OR) of 3.229 [95% confidence interval (CI) = 2.43–4.267] (13). Furthermore, medical evidence supported the notion that SLE is associated with increased susceptibility to malignancies of the female reproductive tract. A total of 302 cases of cervical cancer and 72 cases of vaginal cancer/vulvar cancer were identified among 247,575 patients with SLE (14). In addition, women with SLE have a significantly greater risk of developing precancerous lesions than those without SLE (15).

It is unknown why patients with SLE have a higher incidence of malignancies of the reproductive tract, but this may be attributable to the following: overlap with Sjogren’s syndrome, lupus-associated medications, viral infections, conventional cancer risk factors, and innate immune system abnormalities (11). SLE is characterized by the formation of numerous immune complexes through the binding of autoantibodies (anti-DNA and anti-histone antibodies) to their corresponding autoantigens (16, 17), which are deposited throughout the body, activating the complement system and leading to tissue damage. Patients with SLE have an increased risk of developing malignancies due to the accumulation of DNA damage caused by autoantibodies that interfere with DNA repair (18). In addition, SLE is a chronic, difficult-to-treat disease that can compromise normal organ function and impact multiple organ systems. Moreover, the compromised capacity to combat oncogenic viruses and inherent immune dysfunction may contribute to the increased cancer risk among patients with SLE. Furthermore, age, duration of SLE, and the risk of developing malignancies of the reproductive tract are positively correlated (2).

Patients with SLE frequently require the use of immunosuppressants or corticosteroids, which may promote the development of tumors, lower immunity, and increase the risk of infection (19). When the disease manifests refractory symptoms or impacts major organs, systemic glucocorticoids, cyclophosphamide, methotrexate, or azathioprine are suggested as treatment options (20). These medications may increase the risk of infection, decrease immunity, and promote the development of malignancies. Hsu et al. conducted a study that identified a potential dose-dependent association between cyclophosphamide treatment and the overall elevated risk of malignancy among patients with SLE (21). In addition, the use of hydroxychloroquine may reduce the overall risk of cancer (21, 22), indicating that the impact of drug treatment on the cancer risk in patients with SLE could be complex. Furthermore, an investigation conducted by Wadstrom et al. examined the correlation between exposure to immunosuppressive drugs and the likelihood of developing cervical neoplasia and pre-neoplastic lesions (23). In contrast to patients with SLE who received antimalarial therapy, those who underwent immunosuppressant treatment exhibited a 1.83-fold elevated risk of developing cervical neoplasia [hazard ratio (HR) = 1.83, 95% CI = 1.15–2.91)]. This indicates that the use of immunosuppressants may increase the incidence of cervical cancer among patients with SLE.

Women with SLE may exhibit an increased vulnerability to infection caused by viruses associated with reproductive tract tumors, such as HPV, which has been attributed to the progression of these tumors. Studies have indicated that female SLE patients have a higher rate of infection with high-risk HPV strains and are more likely to be infected with multiple HPV subtypes (24, 25), thereby increasing their risk of HSIL, especially those exposed to immunosuppressants (26). Immunosuppressants may reduce the ability of SLE patients to clear HPV infections (27). Studies have found that the incidence of guideline-recommended cervical cancer screening (CCS) among patients with SLE, even in tertiary hospitals (28, 29), is extremely low; this underscores the need for greater CCS awareness.

SLE-associated synchronous malignant tumors of the reproductive tract have not been documented in the past. However, there is evidence that patients with SLE have an increased risk of developing tumors of the reproductive tract; thus, early detection and consistent monitoring are vital. These tests, however, are easily disregarded, including HPV and TCT. Physicians must increase their focus on CCS to prevent the omission of precancerous lesions. The HPV vaccine is effective and well tolerated in patients with SLE. A recent study has found that immunogenicity is maintained in the majority of patients after 5 years (30). Patients with SLE who also have concurrent tumors are more effectively treated surgically. Treatment can be resumed post-chemotherapy completion without impacting the prognosis of the autoimmune disease. During subsequent chemotherapy, immunosuppressants or steroids should be discontinued. During cancer treatment, assessment of the activity of SLE is also extremely important (31); however, no reports have indicated that the activity of SLE is related to tumor progression. Patients with reproductive tract tumor prognoses are influenced by lymph node metastasis, tumor volume, stage, and vascular stromal infiltration. However, there is still a lack of studies regarding the effects of SLE on survival rates. During a 1-year follow-up period, neither of the two cases of genital tract double cancer in this report exhibited signs of recurrence; therefore, ongoing long-term surveillance is necessary. Subsequent validation using a more extensive sample size is also required.

We present cases of SLE co-occurring with synchronous genital tract malignant tumors for the first time. Physicians should prioritize the reproductive health of female SLE patients and ensure that they receive comprehensive follow-up, as demonstrated by these cases. Routine monitoring and assessment are imperative to promptly identify any potential tumors and concurrent malignancies should elicit apprehension. This uncommon case report offers more comprehensive insights into the correlation between SLE and tumors of the reproductive tract, thereby suggesting possible preventive and therapeutic approaches.

## Data availability statement

The original contributions presented in the study are included in the article/supplementary material. Further inquiries can be directed to the corresponding author.

## Ethics statement

Ethical approval was not required for the study involving humans in accordance with the local legislation and institutional requirements. Written informed consent to participate in this study was not required from the participants or the participants’ legal guardians/next of kin in accordance with the national legislation and the institutional requirements. Written informed consent was obtained from the individual(s), and minor(s)’ legal guardian/next of kin for the publication of any potentially identifiable images or data included in this article.

## Author contributions

LW: Formal Analysis, Methodology, Writing – original draft. NS: Visualization, Writing – original draft. JW: Data curation, Formal analysis, Writing – original draft. SS: Formal Analysis, Writing – original draft. HY: Writing – original draft. QZ: Supervision, Writing – review & editing. XC: Funding acquisition, Methodology, Project administration, Supervision, Writing – review & editing.
